# Volatile Organic Compounds Produced by *Bacillus velezensis* CT32 Attenuate *Botrytis cinerea* Pathogenicity by Impairing Autophagy

**DOI:** 10.3390/jof12100731

**Published:** 2026-10-01

**Authors:** Xinxin Li, Chao Han, Baohua Tian, Ruilin Xiao, Qing Guo, Shiqi Cao, Haixia Liang

**Affiliations:** 1 College of Environment and Ecology, Taiyuan University of Technology, Taiyuan 030024, China; lixinxin@tyut.edu.cn (X.L.);; 2 Changzhi Institute of Science and Technology Innovation and Development Research, Changzhi 046000, China; 3 School of Physical Education and Health Engineering, Taiyuan University of Technology, Taiyuan 030024, China; 4 College of Artificial Intelligence, Taiyuan University of Technology, Taiyuan 030024, China

**Keywords:** *Bacillus velezensis*, gray mold, volatile organic compounds, autophagy, pathogenicity

## Abstract

Gray mold, caused by *Botrytis cinerea*, is the predominant fungal disease responsible for postharvest losses in strawberries. Here, we show that volatile organic compounds (VOCs) released by *Bacillus velezensis* CT32 significantly inhibited *B. cinerea* mycelial growth and attenuated its pathogenicity on strawberry fruit, reducing gray mold incidence by 51.35%. An optimized headspace solid−phase microextraction−gas chromatography−mass spectrometry method was developed to profile CT32 VOCs, identifying 30 compounds, predominantly aldehydes, alcohols, and ketones. Scanning electron microscopy revealed that CT32 VOCs severely disrupted hyphal surface structure, as evidenced by deformation, collapse, peeling, and breakage. Comparative transcriptomic analysis further revealed that exposure to CT32 VOCs significantly downregulated pathogenicity−associated genes and pathways involved in fructose and mannose metabolism, arginine and proline metabolism, proteasome, and autophagy in *B. cinerea*. This transcriptional signature was corroborated by fluorescent labeling and ultrastructural observations, which indicated that CT32 VOCs impaired autophagy in *B. cinerea*. Collectively, strain CT32 VOCs exert antifungal activity through a multi−component, multi−target mode of action that compromises hyphal integrity, perturbs carbohydrate and amino acid metabolism, and disrupts intracellular homeostasis in *B. cinerea*, with autophagy impairment further restricting nutrient recycling and cellular damage clearance and hindering homeostatic recovery, thereby suppressing fungal growth and pathogenicity.

## 1. Introduction

*Botrytis cinerea*, the causal agent of gray mold, is a non-host-specific, necrotrophic fungus that affects over 200 plant species, causing economic damages exceeding USD 10 billion per annum [1]. During recent decades, the rapid emergence and global spread of fungicide-resistant phenotypes have posed a great challenge to effectively control gray mold disease [2]. Management of postharvest gray mold in strawberry (*Fragaria* × *ananassa* Duch.) is particularly challenging due to the delicate nature of fruit, which makes them highly susceptible to fungal infection and prone to contamination with fungicide residues, further limiting the reliance on chemical strategies for handling Botrytis rot in stored strawberries. Addressing this challenge requires the exploration and development of safer, eco-friendly and highly efficient alternatives to minimize postharvest losses, as well as to mitigate the risk of resistance development in *B. cinerea*.

Recent advances in understanding of bacterial volatiles as potent antifungal weapons, long-range communication signals and behavioral modulators have provided unprecedented opportunities for the potential biotechnological application of bacterial volatiles in controlling postharvest decay of fruits [3]. Volatile organic compounds (VOCs) released by bacteria are low-molecular-weight (<300 Da) primary or secondary metabolites characterized by a low boiling point, high vapor pressure, and lipophilic character, which are formed via a variety of biosynthetic pathways, such as the shikimate pathway, fatty acid synthesis and amino acid degradation pathways, etc. [4]. The diverse physicochemical properties and biological activities of these compounds, along with their ease of diffusion both in the gas and water phase, enable them to function effectively as biofumigants, promoters of plant growth and elicitors of fruit resistance [5]. Commonly produced VOCs of *Bacillus* origin are alcohols, acids, esters, aldehydes, ketones, phenols, many of which have been reported to possess broad-spectrum antifungal activities against *Monilinia fructicola*, *Alternaria solani*, *Penicillium expansum*, *B. cinerea*, *P. italicum*, and others [6,7]. Fumigation with either antifungal volatile-producing strains or pure synthetic compounds has proven to be effective in controlling fungal decay in fruits and extending their shelf life [8,9]. For example, postharvest exposure of peaches to volatile blends emitted by *B. subtilis* CF-3 or *B. cereus* N4, as well as synthetic VOCs of their origin such as benzothiazole, 3-methyl-1-butanol, and s-methyl butanethioate, restrained spot expansion and induced fruit resistance to brown rot [10,11]. In contrast to a single synthetic compound, the bacterial volatile blends are biodegradable as naturally occurring substances and can exert their effects at low concentrations. Therefore, bacterial volatiles can be exploited as a sustainable strategy for use in postharvest disease control. However, given the diversity and specificity of endogenous metabolic pathways in bacteria, even within the same species, only a limited number of volatile metabolites have been identified so far, and many more remain to be discovered.

It is well known that bacterial volatiles can antagonize fungal pathogens, but the exact mechanisms of action and molecular targets of this effect are not entirely clear. The physicochemical attributes of bacterial volatiles facilitate their passive diffusion across cellular barriers, such as fungal cell walls or membranes. Consequently, these volatile signals have frequently been reported to inhibit cell wall biosynthesis, interfere with cell wall cross-linking, disrupt membrane integrity, and increase membrane permeability in a number of phytopathogenic fungi, resulting in morphological changes and leakage of cytoplasmic components [12]. Exposure to *Bacillus*-derived VOCs has also been reported to promote reactive oxygen species accumulation [13], impair mitochondrial function [14], and perturb key metabolic pathways in fungal cells [15], thereby disturbing intracellular redox balance and energy homeostasis [16]. Accumulating evidence indicates that bacterial volatiles exert broad-spectrum antifungal activity not by acting on a single cellular component, process, or pathway, but through multiple interconnected mechanisms. Accordingly, deciphering their multi-target, synergistic modes of action requires moving beyond single-scale analyses and integrating VOC profiling with analyses of macroscopic phenotypes, microscopic structures, cellular physiology, and molecular regulation.

To this end, we integrated headspace solid-phase microextraction–gas chromatography–mass spectrometry (HS-SPME-GC-MS) profiling of VOCs produced by *B. velezensis* CT32 with multi-scale analyses of the response of *B. cinerea* to VOC stress, including assays of fungal growth in vitro and pathogenicity on strawberry fruit, observations of cellular morphology and ultrastructure, autophagy monitoring, and transcriptome sequencing, to systematically characterize the antifungal activity of strain CT32 VOCs and elucidate the multi-component, multi-target, multifaceted mode of action by which these VOCs disrupt fungal cellular homeostasis and suppress the growth and pathogenicity of *B. cinerea*. Beyond extending current understanding of volatile-mediated interactions between antagonistic bacteria and pathogenic fungi, this study also highlights the potential of bacterial VOCs as biofumigants for controlling postharvest fungal decay in fruit.

## 2. Materials and Methods

### 2.1. Strains, Media and Culture Conditions

*B. velezensis* CT32 producing antifungal volatiles was preserved in the China Center for Type Culture Collection (CCTCC M 2021995), Wuhan, China. The bacterial strain was grown on a nutrient agar (NA) plate at 28 °C. *B. cinerea* was preserved in the Agricultural Culture Collection of China (ACCC 37712), Beijing, China. The fungal pathogen was grown on a potato dextrose agar (PDA) plate in the dark at 22 °C.

### 2.2. Effect of Strain CT32 VOCs on In Vitro Growth of B. cinerea

A Petri dish containing PDA was inoculated in the center with a 5 mm diameter mycelial plug of *B. cinerea*. The other Petri dish containing NA was evenly spread with 200 µL of strain CT32 suspension (1 × 10^7^ CFU mL^−1^). After removing the lids of both plates, the PDA plate inoculated with fungal pathogen was inverted and placed over the NA plate inoculated with antagonistic bacterium. Subsequently, the two plates were sealed with Parafilm and incubated at 22 °C. In the tightly sealed environment of plates, *B. cinerea* was fully exposed to *B. velezensis* CT32 VOCs (BV). Under the same condition, the PDA plate inoculated with fungus was sealed with the NA plate not inoculated with bacterium and used as a control. The diameter of the fungal colony in each plate was measured when mycelia completely covered the control plates. The experiment consisted of 15 replicates.

The microstructures of mycelia collected from BV-exposed and control plates were observed separately using scanning electron microscopy (SEM). The fungal samples were prefixed in 2.5% glutaraldehyde, rinsed thrice with 0.1 M phosphate buffer, postfixed in 1% osmium tetroxide, rinsed in buffer, and then dehydrated through a graded ethanol series, replaced with isoamyl acetate, critical-point dried and sputter-coated with gold, as described by Tahir et al. [17]. Finally, the specimens were viewed under a SU8010 microscope (Hitachi, Tokyo, Japan).

### 2.3. Effect of Strain CT32 VOCs on Pathogenicity of B. cinerea

Mature fruits of a commercial strawberry cultivar (*Fragaria* × *ananassa* Duch. cv. Benihoppe) that were uninjured, uniform in size and color, and free from pests and diseases were selected. The strawberries were immersed in 2% sodium hypochlorite for 2 min, washed twice with sterile distilled water, and dried naturally. For inoculum preparation, mycelial disks (2 mm in diameter) were taken from the periphery of 7-day-old cultures of *B. cinerea* exposed or not exposed to BV, as described in Section 2.2. For inoculation, a wound (3 mm deep × 2 mm wide) was made on the surface of strawberry fruit. Subsequently, each artificially wounded fruit was placed into a 2 L airtight box with a divider and subjected to one of the following treatments: inoculated with unexposed mycelia (control); inoculated with mycelia pre-exposed to BV (pre-exposure); or inoculated with mycelia and then exposed to BV (post-exposure). In the first two treatments, 2 Petri dishes containing NA were placed at the bottom of the box. In the third treatment, 2 NA plates spread with 200 µL of strain CT32 suspension (1 × 10^7^ CFU mL^−1^) were placed at the bottom of the box. Each treatment comprised three replicates, and each replicate consisted of 9 strawberries. The experiment was conducted thrice. These sealed boxes with infected fruits were kept at 22 °C and examined for disease incidence and lesion diameter at 5 days post inoculation (dpi). Disease severity of gray mold on strawberries was assessed according to the area of lesions, which gave infected tissue a decayed, necrotic, grayish, powdery appearance, and was rated on a scale modified from Russi et al. [18]: 0, healthy fruit; 1, 1–25% area affected; 2, 26–50% area affected; 3, 51–75% area affected; 4, 76–100% area affected. Disease index was calculated using the formula given by Li et al. [19].

### 2.4. Analysis of Strain CT32 VOCs by HS-SPME-GC-MS

#### 2.4.1. Sample Preparation

A 20 mL headspace vial containing 3 mL of melted NA medium was sterilized at 121 °C for 20 min. After solidification, the surface of the culture medium was inoculated with 90 µL of strain CT32 cell suspension (1 × 10^7^ CFU mL^−1^). The vial was tightly closed with a polytetrafluoroethylene/silicone septum and incubated for 4 d at 28 °C. Samples were prepared in triplicate, and uninoculated vials containing only the culture medium served as controls.

#### 2.4.2. HS-SPME Method Development

The experimental procedure for VOC profiling of strain CT32 is illustrated in Figure S1. First, five types of SPME fibers coated with different stationary phases (Supelco, Bellefonte, PA, USA), 50/30 µm divinylbenzene/carboxen/polydimethylsiloxane (DVB/CAR/PDMS), 65 µm polydimethylsiloxane/divinylbenzene (PDMS/DVB), 100 µm polydimethylsiloxane (PDMS), 75 μm (CAR/PDMS), and 85 μm polyacrylate (PA), were tested for determining which fiber would be the most adequate for headspace sampling of bacterial VOCs. The vial was positioned inside a water bath to equilibrate for 20 min at 70 °C, and then sampling was carried out by exposing the SPME fiber to the headspace of the bacterial culture for 40 min. The trapped compounds were desorbed by inserting the fiber into the GC injection port for 4 min at 250 °C. The overall extraction performance of each fiber for bacterial VOCs was assessed based on the number and total peak area of the chromatographic peaks. Subsequently, the effects of extraction temperature (50, 60, 70, 80 and 90 °C), extraction time (30, 40, 50, 60 and 70 min), and equilibrium time (10, 20, 30, 40 and 50 min) on the extraction efficiency of bacterial VOCs using an optimal SPME fiber were investigated by single-factor experiments.

To maximize the number of bacterial VOCs that can be adsorbed onto the selected fiber, a three-factor Box–Behnken design (BBD) based on response surface methodology (RSM) was used to investigate the impact of three variables, namely extraction temperature, extraction time and equilibrium time, on the number of peaks (NPs) as the response. Three levels of −1, 0, and +1 were considered for each variable, and the range of values were chosen based on the optimum conditions obtained in the single-factor experiments. A quadratic polynomial equation was used to construct the response surface model:

$$y=\beta_{0}+\sum_{i=1}^{k}\beta_{i}x_{i}+\sum_{i=1}^{k}\beta_{ii}{x_{i}}^{2}+\sum_{1\leq i\leq j}^{k}\beta_{ij}x_{i}x_{j}+\varepsilon$$

where *y* is the response, *x_i_* and *x_j_* are the variables, *β_0_* is the constant, *ε* is the residual, and *β_i_*, *β_ij_* and *β_ii_* represent the coefficients of the linear terms, interaction terms, and quadratic terms, respectively.

#### 2.4.3. GC-MS Analysis

GC-MS analysis was carried out on a 7890A gas chromatograph coupled to a 5975C mass selective detector (Agilent, Santa Clara, CA, USA). Chromatographic separation was performed on an HP-5MS capillary column (30 m × 0.25 mm ID × 0.25 μm) with a helium flow rate of 1 mL min^−1^. The temperature of injector was set at 250 °C and operated in splitless mode. The oven temperature program (total run time of 68.5 min) is as follows: start at 40 °C for 2 min, ramped to 110 °C at 4 °C min^−1^, ramped to 130 °C at 2 °C min^−1^ (held for 2 min), ramped to 150 °C at 2 °C min^−1^ (held for 2 min), ramped to 200 °C at 3 °C min^−1^, and finally ramped to 300 °C at 30 °C min^−1^ and held at this temperature for 5 min. Ionization mode: Electron impact at 70 eV. Acquisition mode: Full scan (33–500 *m*/*z*). Ion source and quadrupole temperatures: 230 °C and 150 °C, respectively. The retention index (RI) of each volatile compound was determined by analyzing a standard mixture of *n*-alkanes (C_7_–C_40_) under identical chromatographic conditions and was calculated according to the equation proposed by Bianchi et al. [20]. Bacterial VOCs were qualitatively identified by comparing their mass spectra and RIs to those stored in the NIST 11 Mass Spectral Library or to those reported in the literature. The relative abundances (RA) of the detected volatile components were calculated by the peak area normalization method. The analysis of the NA medium was performed under the same conditions.

### 2.5. RNA Preparation, cDNA Library Construction and Transcriptome Sequencing

Mycelia of *B. cinerea* exposed or not exposed to BV were prepared according to the method described in Section 2.2 and served as the BV group and CK group, respectively. Three samples were prepared in parallel for each group. Fungal mycelia of each sample were harvested from three biological replicates and mixed for RNA extraction. The mycelial samples were rapidly frozen in liquid nitrogen and stored at −80 °C. A total of 100 mg of mycelium was used for RNA extraction per sample. Briefly, RNA preparation, library construction and transcriptome sequencing were accomplished by Majorbio Bio-Pharm Technology Co., Ltd. (Shanghai, China) using an Illumina NovaSeq X Plus platform. The raw RNA-seq data have been deposited in Sequence Read Archive under the accession number: PRJNA1171356.

### 2.6. Bioinformatics Analysis of Transcriptome Data

Raw data were quality-controlled by filtering out low-quality reads, as well as reads containing poly-N or adapter to get high-quality clean data [21]. Subsequently, the clean reads were aligned to the genome database of *B. cinerea* strain B05.10 using HISAT2 (version 2.1.0) with outFilterMismatchNmax set to 2. To evaluate gene expression levels, read counts of each transcript were normalized to fragments per kilobase per million fragments mapped (FPKM) using RSEM (version 1.3.3). Differentially expressed genes (DEGs) were screened with DESeq2 (version 1.24.0) and identified if their expressions significantly changed between the CK group and BV group. The criterion for detecting alteration in gene expression was an adjusted *p*-value < 0.01 and an absolute value of log_2_ fold-change ≥ 1. Functional annotation and enrichment analysis of the candidate DEGs were performed based on the Gene Ontology (GO) and Kyoto Encyclopedia of Genes and Genomes (KEGG) databases.

### 2.7. RT-qPCR Validation

Mycelia exposed or not exposed to BV were ground separately in liquid nitrogen. Fungal RNA was extracted from 100 mg of frozen ground mycelia using an E.Z.N.A. Fungal RNA Kit (Omega, Norcross, GA, USA) following the manufacturer’s instructions. Then RNA concentration was quantified using a NanoVue Plus Spectrophotometer (Biochrom, Cambridge, UK). RNA was reverse-transcribed into cDNA using a GoScript Reverse Transcription System (Promega, Madison, WI, USA). A total of 1 μg RNA was mixed with 1 μL random primers and 1 μL oligo(dT)_15_ primer, and nuclease-free water was added to a total volume to 10 μL. The mixture was pre-denatured at 70 °C for 5 min and chilled on ice. A total of 10 μL RT-Mix was then added to a final volume to 20 μL, and reverse transcription was performed under the following conditions: 25 °C for 5 min; 42 °C for 60 min; and 70 °C for 15 min. The cDNA was diluted 10-fold prior to qPCR. qPCR reactions were run with SGExcel FastSYBR qPCR Mixture (Sangon Biotech, Shanghai, China) on the CFX96 Real-Time System (Bio-Rad, Hercules, CA, USA). The 20 μL qPCR reaction mixture consisted of 2 μL cDNA, 0.4 μL forward primer (10 μM), 10 μL SGExcel FastSYBR mixture, 0.4 μL reverse primer (10 μM), and 7.2 μL RNase-free ddH_2_O. qPCR amplification procedure was performed as follows: pre-denaturation at 95 °C for 3 min; and 40 cycles of 95 °C for 5 s and 60 °C for 20 s. Relative expression levels of genes were determined via the 2^−ΔΔ^*^C^*^T^ method reported by Livak and Schmittgen [22], with BcActin as an internal reference gene. Gene-specific primers were designed, and their sequences are listed in Table S1. The experiment was performed in triplicate.

### 2.8. Analysis of Autophagy by Fluorescent Staining and TEM Observation

Fungal mycelia were collected from BV-exposed and control plates, respectively, as described in Section 2.2. To monitor the starvation-induced autophagy, mycelia from each plate were transferred into a nitrogen-limiting medium (MM-N) containing 2 mM phenylmethylsulfonyl fluoride (PMSF) and incubated at 22 °C and 180 rpm for 6 h, according to the method given by Ren et al. [23]. Subsequently, fungal samples kept in normal culture condition or starved for 6 h were stained using a Monodansylcadaverine (MDC) Staining Kit (Solarbio, Beijing, China) and observed under a Lionheart FX automated microscope (BioTek, Winooski, VT, USA) to detect autophagy. Moreover, the ultrastructure of nitrogen-starved mycelia of *B. cinerea* exposed or not exposed to BV was observed under an HT7700 microscope (Hitachi, Tokyo, Japan) after a series of steps including fixation, dehydration, embedding, sectioning, and staining.

### 2.9. Statistical Analysis

Design-Expert software (version 8.0.6) was employed to obtain the polynomial regression model of the response surface to estimate the optimum conditions for maximum extraction of bacterial VOCs. Analysis of variance (ANOVA) was used to assess the statistical significance of the model developed. Data were calculated and subjected to one-way ANOVA or Student’s *t*-test using SPSS software (version 21.0).

## 3. Results

### 3.1. BV Suppress the Mycelial Growth of B. cinerea In Vitro

*B. cinerea* was highly susceptible to VOCs emitted by strain CT32; the mycelia were sparse and partially dissolved following exposure to BV compared to the control (Figure 1A). In addition, BV exposure significantly reduced the mycelial growth of the phytopathogen, with an inhibition rate of 43.17% (Figure 1B). Additionally, SEM observation revealed distinct alterations in the morphology of mycelia exposed to BV, with rough, shriveled, twisted, and collapsed surfaces, while the appearance of the control mycelia remained plump and smooth (Figure 1C).

### 3.2. BV Attenuate the Pathogenicity of B. cinerea Towards Strawberry Fruits

The symptoms of grayish and fuzzy spots were initially observed on strawberry fruits inoculated with unexposed mycelia at 2 days post inoculation (dpi); the disease incidence and index reached maximum values of 91.36% and 33.33 at 5 dpi, respectively (Figure 1D). Strawberries inoculated with mycelia pre-exposed to BV significantly reduced disease incidence by 51.35% compared to fruits inoculated with unexposed mycelia (Figure 1E). BV exposure after inoculation effectively alleviated symptoms of gray mold rot in strawberries during storage, with a disease index of 22.84 (Figure 1F). Collectively, exposure of *B. cinerea* to BV was effective in controlling gray mold disease by attenuating pathogenicity of the necrotrophic fungal pathogen towards postharvest strawberries.

### 3.3. HS-SPME-GC-MS Approach to Characterize the Volatile Profile of Strain CT32

#### 3.3.1. Preliminary Study of HS-SPME Parameters by Single-Factor Experiments

To improve the performance of the HS-SPME method, preliminary experiments were conducted to investigate the influence of individual parameters on the extraction efficiency of bacterial VOCs while keeping other parameters constant. Five types of SPME fibers coated with different stationary phases were evaluated in terms of their capacity to extract BV (Figure 2A). The overall performance of each fiber was assessed according to the number and total area of chromatographic peaks. The triphasic DVB/CAR/PDMS fiber was the most efficient at adsorbing trace VOCs in the headspace of the bacterial culture. During the extraction process, BV migrate between the culture matrix, headspace and fiber coating until equilibrium is achieved. As depicted in Figure 2B, the NPs increased with a rising temperature of extraction from 50 °C to 70 °C. Then NPs reduced significantly when the temperature rose to 80 °C, while the total peak area slowly increased. This is due to the fact that elevating temperature can enhance the diffusion rates of analytes, which favors evaporation of VOCs from the bacterial culture into the gaseous phase. As a consequence, the rate of volatile compound transfer onto the fiber coating and the sensitivity of headspace analysis are improved. Nevertheless, from the thermodynamic viewpoint, excessive heating of the sample reduces the coating/gas distribution constant, since SPME extraction is an exothermic process, thereby reducing adsorption of target analytes onto the fiber coated with DVB/CAR/PDMS. Accordingly, the extraction temperature was fixed at 70 °C. As illustrated in Figure 2C, both the NPs and total peak area dramatically increased at first, reached the maximum at 50 min, and then slightly decreased as the extraction time prolonged to 60 min. This indicated that an extraction time of 50 min was sufficient for BV to attain distribution equilibrium between the headspace and the fiber coating. A further increase in extraction time may lead to replacement of weakly bonded compounds by volatiles that are more affined to the fiber sorbents. As depicted in Figure 2D, the amount of analytes adsorbed was significantly increased with the prolongation of equilibrium time and tended to be stable after 20 min. This means that once the volatile metabolites achieve equilibrium between the headspace and bacterial culture, the amount extracted is independent of further increases in equilibrium time. Hence, an equilibrium time of 20 min was adopted.

#### 3.3.2. Optimization of HS-SPME Condition by Response Surface Methodology

Taking into account the interactive effects among the parameters studied, HS-SPME condition was further optimized with a BBD of RSM to maximize the number of compounds that could be adsorbed onto the DVB/CAR/PDMS-coated fiber. The matrix for BBD consisted of 17 experimental runs performed in random order, as summarized in Table S2. Using the experimental data obtained, the following response surface model was generated:

*y* = 48.80 + 3.13*x*_1_ + 1.25*x*_2_ + 0.63*x*_3_ + 1.00*x*_1_*x*_2_ + 0.25*x*_1_*x*_3_ − 4.53*x*_1_^2^ − 2.78*x*_2_^2^ − 0.52*x*_3_^2^

where *y* is the number of peaks, *x*_1_ signifies the extraction temperature, *x*_2_ signifies the extraction time, and *x*_3_ signifies the equilibrium time.

Further, the significance and adequacy of the mathematical model proposed by RSM were statistically assessed by ANOVA (Table 1). The coefficient of determination (R^2^) and adjusted R^2^ were 0.9932 and 0.9844 respectively, while the lack of fit (*p* = 0.4028) was not significant. This indicated that there was a good fit between the established model and experimental data. The *F*-value of the model was 113.51, and the *p*-value was below 0.0001. These results suggested that the model was reliable and effective in predicting the peak counts of bacterial VOCs. The *p*-values of *x*_1_, *x*_2_, and *x*_3_ were less than 0.01, indicating that all chosen parameters had significant effects on the extraction process. The quadratic terms *x*_1_^2^ and *x*_2_^2^ were highly significant (*p* < 0.0001), implying that the NPs response was extremely influenced by extraction temperature and extraction time. The significance level of *x*_1_*x*_2_ in terms of *p*-value was 0.0038, demonstrating that the impact of the two parameters upon the extraction efficiency was interdependent. According to *F*-values from high to low, the influence of the three process parameters on the NPs response ranked as follows: extraction temperature > extraction time > equilibrium time.

Figure 2E,F depict the effect of extraction temperature, extraction time and their combination on NPs at a fixed equilibrium time of 20 min. The shape of the 2D plot is elliptical, and the slope of the 3D diagram is steep, implying significant interaction between the corresponding variables. Figure 2G,H reflect the presence of maximum adsorption amount of BV by DVB/CAR/PDMS-coated fiber. When the extraction time was kept at 50 min, extraction temperature and equilibrium time that provided maximum NPs for BV were 73.98 °C and 26.89 min, respectively. It can be seen from Figure 2I,J that the interactive effect of extraction time and equilibrium time has little impact on the NPs. The extraction of VOCs from bacterial emissions was best achieved at an extraction and equilibrium time of 52.97 min and 26.89 min, respectively, when extraction temperature was held at 0 level (70 °C).

As predicted by the RSM regression model, the maximum NPs was 49.8216 at HS-SPME condition of a 73.98 °C extraction temperature, a 52.97 min extraction time, and a 26.89 min equilibrium time. Considering the operational aspects, 74 °C, 53 min, and 27 min were adopted as a compromise condition. To further verify the reliability of the quadratic model in predicting the response value, validation tests were performed in triplicate under the optimized extraction condition adopted above. The experimentally determined value (NPs of 50) was basically consistent with the theoretical value (NPs of 49.8216) calculated by the software, thus indicating that the developed HS-SPME-GC-MS method for profiling volatile components produced by strain CT32 is suitable, effective and feasible.

#### 3.3.3. Analysis of Chemical Composition of BV by HS-SPME-GC-MS

A total number of 50 effective peaks were detected in the total ion chromatogram of BV, of which 30 were identified by GC-MS, as depicted in Figure 2K,L. These compounds were classified into 10 categories according to their functional groups, namely four aldehydes, five alcohols, six ketones, eight alkanes, one ester, two phenols, one alkene, one nitrogen compound, one biphenyl, and one thiazole (Table S3). The most abundant categories of volatile metabolites in relative abundances were aldehydes, alcohols and ketones, which accounted for 50.32%, 24.31% and 11.28% of the total volatile components, respectively. Dodecanal (48.56%) was discovered as a predominant constituent of BV, followed by 1-dodecanol (10.27%) and 2-undecanol (8.46%), while octadecane (0.05%), benzothiazole (0.06%) and 4,7-dimethylundecane (0.09%) were identified as trace constituents emitted from strain CT32.

### 3.4. Transcriptome of B. cinerea Is Altered by BV Treatment

To elucidate the molecular mechanism underlying the response of *B. cinerea* to bacterial VOC stress, fungal mycelia were harvested from BV-treated and the control pathogen and subjected to transcriptome analysis. Each sample generated an average of 6.48 Gb clean bases, of which the percentage of Q30 bases was higher than 94.39% (Table S4). GC content of all samples exceeded 45.90%. Between 65.35% and 96.04% of clean reads were successfully mapped to the genome of the reference strain B05.10. These results indicated that the quality of the *B. cinerea* RNA-seq data was sufficient for subsequent bioinformatic analysis.

A total of 2328 transcripts differed significantly between the fungal pathogens from BV-exposed group and control group, including increases in 1157 genes and decreases in 1171 genes, respectively (Figure 3A). Functional annotation of DEGs in *B. cinerea* subjected to BV stress was accomplished using the GO database. As depicted in Figure 3B, a total of 2328 DEGs were successfully assigned to 39 functional groups, belonging to three major categories. In the cellular component category, the most highly enriched terms were “membrane part” and “membrane”, followed by “cell part”, demonstrating that the structure or formation of the fungal membrane changed during the volatile-mediated interaction between *B. cinerea* and *B. velezensis*. In the molecular function category, GO terms were mainly enriched in “catalytic activity”, “binding”, “transporter activity” and “antioxidant activity”. In the biological process category, “metabolic process” and “cellular process” were the predominantly enriched GO terms for DEGs. As expected, GO terms related to “response to stimulus”, “biological regulation” and “detoxification” were also overrepresented, suggesting that *B. cinerea* could sense chemical signals of bacterial origin and regulate proper responses in the cell to mitigate or repair the damage caused by BV, thereby maintaining fungal survival.

GO enrichment analysis further revealed that DEGs were significantly enriched in molecular functions associated with “oxidoreductase activity” and “catalytic activity” and were all downregulated in “proteasomal ubiquitin-independent protein catabolic process”, as well as in cellular components related to “proteasome complex” and “proteasome storage granule” (Figure 3C). To explore possible connections between genes and metabolic and signal transduction pathways, KEGG pathway analysis of downregulated DEGs was performed (Figure 3D). A large proportion of DEGs were significantly enriched in the carbohydrate metabolic pathway and amino acid metabolic pathway, which indicated the involvement and importance of carbohydrate and amino acid metabolism in regulating the responses of the fungal pathogen to BV stimulation. Compared with the CK group, DEGs that decreased in the BV group were also dramatically enriched in the “proteasome” and “autophagy” pathways. This suggests that BV disrupt protein homeostasis in *B. cinerea* via dual inhibition of autophagic and proteasomal degradation, which are two main protein degradation machineries, thereby leading to defects in fungal growth, stress resistance, and pathogenicity.

### 3.5. BV Repress Transcription of Genes Involved in B. cinerea Pathogenicity

To gain insight into the molecular basis of how BV fumigation controls gray mold caused by fungal infestation, we analyzed the relative expression levels of genes encoding secondary metabolites or proteins required for fungal pathogenicity. Among the two secondary metabolite gene clusters involved in biosynthesis of the phytotoxic compounds botcinic acid and botrydial, more than 90% of the genes showed lower transcript abundance after exposure to BV, as illustrated in Figure 4A,B. The virulence−related genes that exhibited a significant decrease in expression in the BV−exposed fungus when compared to the untreated control also included genes encoding endopolygalacturonases (*BcPG3*, *BcPG4*, *BcPG6*) with plant cell wall degrading activity and the Dicer−like protein (*BcDCL1*) with a function in producing sRNA effectors, ATP−binding cassette (ABC) transporters (*BcatrA*, *BcatrB*, *BcatrO*), and phosphoenolpyruvate carboxykinase (*BcPCK1*) in gluconeogenesis (Figure 4C). RT−qPCR validation of six of these candidate pathogenicity−related genes, i.e., *BcBOT1*, *BcBOA1*, *BcBOA8*, *BcPG4*, *BcDCL1*, and *BcatrO*, confirmed the reliability of RNA−seq data (Figure 4D).

### 3.6. BV Suppress Expression of Autophagy-Related Genes and Formation of Autophagic Structures in B. cinerea

Autophagy in filamentous fungi delivers cytoplasmic components to vacuole for degradation and recycling to maintain cell survival under harsh conditions (Figure 5A). Here we investigated related genes in RNA-seq data to better understand how BV manipulate the autophagy process in *B. cinerea*. It was noteworthy that all 23 autophagy-related (ATG) genes showed a consistent downward trend in BV-exposed fungus compared to the untreated control, among which *BcATG3*, *BcATG5*, *BcATG8*, *BcATG13*, *BcATG16*, and *BcATG23* were significantly downregulated (Figure 5B). RT-qPCR analysis further confirmed that BV exposure significantly reduced the transcript levels of *BcATG3*, *BcATG5*, *BcATG8*, *BcATG13*, and *BcATG16* (Figure 5C). *BcATG13* is a core component of the ATG1 complex, whereas *BcATG3*, *BcATG5*, *BcATG8*, and *BcATG16* function in the ubiquitin-like conjugation systems. These results suggest that BV suppress ATG gene expression.

For monitoring autophagy at the microscopic level, fungal cells were stained with monodansylcadaverine (MDC), which accumulates in acidic compartments, including autophagic vesicles. Under bright-field microscopy, control mycelia undergoing starvation showed the existence of several translucent vacuole-like structures in cytoplasm, consistent with starvation-induced autophagy in the gray mold fungus (Figure 5D). By contrast, no such structures were observed in BV-exposed mycelia during both starvation and nutrient-replete conditions. Consistently, under fluorescence microscopy, numerous MDC-positive structures were observed in control starved mycelia, whereas no fluorescent signal was detected in BV-exposed mycelia. Given its limited specificity, MDC staining alone does not provide definitive evidence of autophagy and therefore should be complemented by ultrastructural analysis. Under nitrogen starvation, the autophagic structure of fungal mycelia in BV-exposed and control groups was observed by transmission electron microscopy (TEM). The starvation-induced accumulation of autophagic bodies observed in unexposed control mycelia was not observed in BV-exposed mycelia (Figure 5E). These results corroborate that BV negatively regulate the transcription of autophagy-related genes, resulting in impairment of autophagic degradation in *B. cinerea*.

## 4. Discussion

Multiple and multidrug resistance of *B. cinerea* to fungicides is a real challenge in sustainable strawberry production, particularly in managing postharvest losses due to Botrytis fruit rot [24]. Most studies have focused on the direct application of antagonists of the genus *Bacillus* or *Saccharomyces* to colonize fruit surface, thereby reducing pathogen load, controlling fungal infection, minimizing rot development, and consequently extending their shelf life [25]. In contrast to these approaches that rely on direct contact with the pathogenic fungus, our study evidenced that biofumigation with volatile metabolites emitted by *B. velezensis* CT32 not only reduced vegetative growth of the filamentous fungus by 43.17%, but also attenuated its pathogenicity, leading to approximately 50% reductions in both the incidence and index of strawberry gray mold disease. The inhibition rates obtained under the present experimental conditions reflect the combined effects of VOC production, headspace volume, and exposure duration. Therefore, further studies should optimize the culture medium, including supplementation with precursor molecules such as amino acids and fatty acids, to promote the biosynthesis of specific antifungal VOCs and thereby enhance the inhibitory activity of CT32 VOCs against *B. cinerea* [26]. In addition, the development of controlled- or sustained-release formulations, together with their incorporation into active packaging systems, may help maintain effective concentrations of bacterial VOCs over extended storage periods, thereby improving their persistence. Reducing the headspace volume and improving sealing could further enhance the fumigation efficacy of bacterial VOCs against strawberry gray mold. Fungicides act as potent inhibitors of fungal reproduction and growth by binding to β-tubulin to block microtubule assembly, inhibiting methionine biosynthesis, or interfering with respiration in plant pathogenic fungi [24], whereas the antifungal activity of BV is achieved by targeting the reduction in pathogenicity rather than directly killing the air-borne fungus. The strategic shift from “killing” to “disarming” the pathogen is expected to alleviate the intense selective pressure imposed by continuous exposure to chemical fungicides, thereby mitigating the risk of resistance development in *B. cinerea*.

The biological activities of VOCs derived from bacteria are contingent upon their physico-chemical properties, such as volatility, polarity and reactivity, which determine how they interact with and affect the phytopathogenic fungi. The volatile profile of strain CT32 encompasses a variety of different classes of compounds, including aldehydes, alcohols, ketones, alkanes, esters, phenols, alkenes, nitrogen compounds, biphenyl, and thiazole. However, the predominant classes of compounds detected in other strains of *B. velezensis* were terpenes, alcohols, alkanes, acids, and ketones [27,28]. This is because, in addition to inconsistencies in sampling and detection methods across diverse studies, the type and relative abundance of VOCs produced by bacteria are influenced by a complex interplay of factors, including medium composition, culture conditions, incubation duration and strain-specific endogenous metabolic pathways responsible for VOC synthesis [29]. Although HS-SPME-GC-MS has been widely used to characterize *Bacillus*-derived VOCs, accurate quantification remains challenging. The data obtained in this study are based on normalized peak areas and therefore only reflect the relative abundances of the detected volatile components. Future work will explore the use of HS-SPME-GC-MS with matrix-matched calibration and stable isotope-labeled internal standards for the targeted absolute quantification of key bacterial volatile compounds. Long-chain aliphatic alcohols with 11–14 carbon atoms identified in the headspace of strain CT32 by HS-SPME-GC-MS are primarily synthesized through fatty acid metabolism by microbial cell factory [30,31]. In addition, secondary alcohols have greater hydrophobicity and lower boiling points than primary alcohols with the same carbon chain length, which makes them more effective in penetrating the lipid bilayer of fungal cell membrane and disrupting membrane fluidity and integrity [32]. Medium-chain aldehydes, such as octanal, nonanal and decanal, due to their high reactivity with nucleophilic groups in the membrane, were reported to damage cell membrane integrity, thereby inhibiting the spore germination and mycelial growth of *Aspergillus flavus* [33]. Previous studies have demonstrated, at both the transcriptional and protein levels, that decanal inhibits *P. expansum* growth and patulin production by disturbing oxidative phosphorylation [34,35]. Benzothiazole exhibits potent inhibition of a wide range of postharvest pathogens, such as *B. cinerea*, *Colletotrichum gloeosporioides*, and *Phytophthora capsici*, *A. flavus* and serves as a privileged scaffold in medicinal chemistry to synthesize new antifungal agents with enhanced efficacy and reduced toxicity by allowing structural modifications to target specific biological pathways [36,37,38]. The multifaceted mechanisms of action of this compound include disruption of energy metabolism, interference with the glyoxylate cycle, suppression of stress responses and metabolic detoxification, and induction of fungal apoptosis [39,40]. Given the synergistic and/or additive antifungal effects exerted by the various components in the volatile blend produced by strain CT32, this biofumigant of bacterial origin primarily targets the cell membrane while also penetrating it to target intracellular components, thereby inhibiting *B. cinerea* growth by concurrently targeting multiple essential cellular processes.

As expected, under the synergistic and/or additive effects of various components in the natural emissions of strain CT32, morphologic changes such as roughening, shriveling, twisting, and breakage were observed on the surface of *B. cinerea* mycelia. These changes suggest that BV may have disrupted cell wall integrity, as well as membrane fluidity or permeability, thereby allowing their entry into fungal cells and interfering with normal physiological functions of *B. cinerea*. This speculation was confirmed at the transcriptional level, as a large number of genes with altered expression in the fungus after continuous exposure to BV were annotated with GO terms related to membrane. Previous studies have suggested that *Bacillus* VOCs interfere with the biosynthesis of chitin, ergosterol, and unsaturated fatty acids, which are key components of the fungal cell wall and membrane [41]. It is noteworthy that GO terms related to “response to stimulus”, “biological regulation”, “detoxification”, and “antioxidant activity” are also overrepresented, suggesting that *B. cinerea* can perceive chemical signals of bacterial origin and regulate the detoxification process and antioxidant defense to resist the adverse effects of BV to some extent.

However, this adaptive response to chemical stress induced by BV comes at the expense of fungal pathogenicity. A previous study found that long-term exposure to VOCs produced by *B. amyloliquefaciens* T-5 weakened *Ralstonia solanacearum* virulence through fitness trade-offs, leading to an almost complete loss of pathogenicity in planta [42]. Our transcriptome sequencing and expression analysis by RT-qPCR further revealed that BV significantly downregulated a comprehensive set of genes encoding phytotoxins or proteins required for fungal pathogenicity, supporting the previous conclusion of in vivo pathogenicity attenuation in *B. cinerea* caused by pre-exposure to BV. Proteins encoded by these genes have been demonstrated to play a multifaceted role in *B. cinerea* pathogenesis by depolymerizing pectin backbone in plant cell wall and silencing host immunity genes via RNA interference [43]. *B. cinerea* knockout mutants for pathogenicity-related genes, such as *BcDCL1*, *BcPCK1*, were defective in conidiation and germination, as well as in penetration of host cells and induction of plant hypersensitive response, and showed reduced lesion development on fruits and vegetables [44,45]. In particular, BV inhibited the fungal secretion of phytotoxins that non-specifically kill host cells by downregulating most genes in the botcinic acid or botrydial biosynthetic gene cluster [46]. Moreover, *BcatrA*, *BcatrB*, and *BcatrO* genes, which encode ATP-binding cassette (ABC) transporters that function as both virulence factors and efflux pumps in *B. cinerea*, also demonstrated significantly downregulated expression in response to BV, implying that the efflux of exogenous BV from fungal cells is greatly impaired. This may lead to increased susceptibility of the fungus and excess accumulation of fungitoxic compounds inside cells, thereby highlighting the potential use of BV as a biofumigant in combating gray mold disease, as well as in overcoming multidrug resistance in *B. cinerea* caused by overexpression of ABC transporter genes.

Autophagy is indispensable for the pathogenicity of fungal pathogens, by participating in differentiation and development, infection structure formation, host penetration, production and deployment of virulence factors, stress adaptation, and plant tissue colonization, all of which are vital for successful infection and disease progression [47,48,49,50,51]. Using comparative transcriptomics, we found that all 23 ATG genes were downregulated in BV-exposed fungus relative to the untreated control, with six genes showing significant downregulation. *BcATG13* is a core component of the ATG1 complex and is therefore involved in autophagy initiation. *BcATG3* and *BcATG8* function in the Atg8 lipidation machinery, whereas *BcATG5* and *BcATG16* are components of the Atg12-Atg5-Atg16 complex, which promotes phagophore expansion [52]. The downregulation of these ATG gene transcripts following BV exposure was accompanied by the loss of detectable MDC fluorescence and the absence of autophagic structures in TEM images, suggesting an association between BV exposure and impaired fungal autophagy. Even under conditions of autophagy induction by starvation, MDC-positive structures were absent from BV-exposed mycelia. Notably, because MDC can also label other acidic compartments, such as late endosomes, these observations provide supportive rather than definitive evidence of altered autophagic activity. Ultrastructural analysis by TEM provided further evidence, revealing the starvation-induced accumulation of autophagic bodies in control mycelia, whereas no such autophagic structures were observed in BV-exposed mycelia. However, several studies have reported that a single volatile compound derived from microorganisms, such as 2-phenylethanol, 2,3-butanedione, or 3-octanol, inhibits fungal growth and controls gray mold disease in postharvest fruits by inducing autophagy of *B. cinerea* [53,54,55], whereas a recent study has demonstrated that ebselen inhibits *B. cinerea* autophagy through targeted inhibition of ATG4 cysteine protease-mediated cleavage of ATG8 [56]. This autophagy inhibition attenuates the pathogenicity of *B. cinerea* on fruits and leaves of several host plants. This inconsistency may arise from the fact that autophagy is primarily cytoprotective, yet excessive or impaired autophagy can also be detrimental to fungal cells [57]. As fungi develop autophagy as an adaptive mechanism towards chemical stress, inhibition of autophagy leads to enhanced susceptibility to bacterial-derived VOCs and accelerates the accumulation of damaged organelles, aggregated proteins, and oxidized lipids, further disrupting intracellular homeostasis and ultimately leading to defects in fungal growth, differentiation, and overall pathogenicity [58]. Extensive studies have revealed that autophagy is required for fungal pathogenicity [59]. Deletion of single autophagy-related genes, such as *BcATG3*, *BcATG4*, *BcATG6*, *BcATG7*, or *BcATG8*, in *B. cinerea* effectively blocks autophagy [23]. The resulting autophagy-deficient mutants exhibit pronounced defects across multiple stages of the fungal life cycle and display markedly attenuated pathogenicity [60,61,62]. Autophagy inhibitors, such as ebselen and its analogs that interfere with autophagosome formation, can suppress hyphal development, spore germination, appressorium formation, and pathogenicity of several phytopathogenic fungi, including *M. fructicola*, *B. cinerea*, and other important Ascomycete pathogens [56]. Our findings reveal a novel action mechanism whereby BV attenuate *B. cinerea* pathogenicity by impairing fungal autophagy. Altogether, this molecular mechanism demonstrates bacterial VOCs as a potential source of natural autophagy modulators. Beyond extending the current understanding of volatile-mediated distant chemical interactions between antagonistic bacteria and pathogenic fungi, these findings highlight the immense promise of targeting autophagy to sustainably control postharvest fungal decay in fruit.

## 5. Conclusions

This study demonstrates that strain CT32 produces 30 chemically distinct volatile metabolites that, through multi-component synergy, compromise the surface integrity of *B. cinerea*, thereby enabling entry into fungal cells, interfering with carbohydrate and amino acid metabolism, inhibiting ABC efflux pumps, and disrupting intracellular homeostasis. Most decisively, BV block autophagic degradation, thereby depriving *B. cinerea* of the compensatory capacity to repair damage and restore homeostasis, while limiting the metabolic precursors and energy supply required for the synthesis of phytotoxins and other virulence- or pathogenicity-associated factors. This multi-target mode of action of BV culminates in profound reductions in fungal fitness, growth and pathogenicity, providing new mechanistic insights into volatile-mediated suppression of gray mold disease. Future research should focus on developing controlled-release formulations, active packaging systems, and biofumigation strategies that modulate the release kinetics and maintain effective antifungal concentrations of bacterial volatiles, thereby advancing their practical application in controlling fungal decay during the postharvest preservation of fresh fruit.

## Figures and Tables

**Figure 1:**
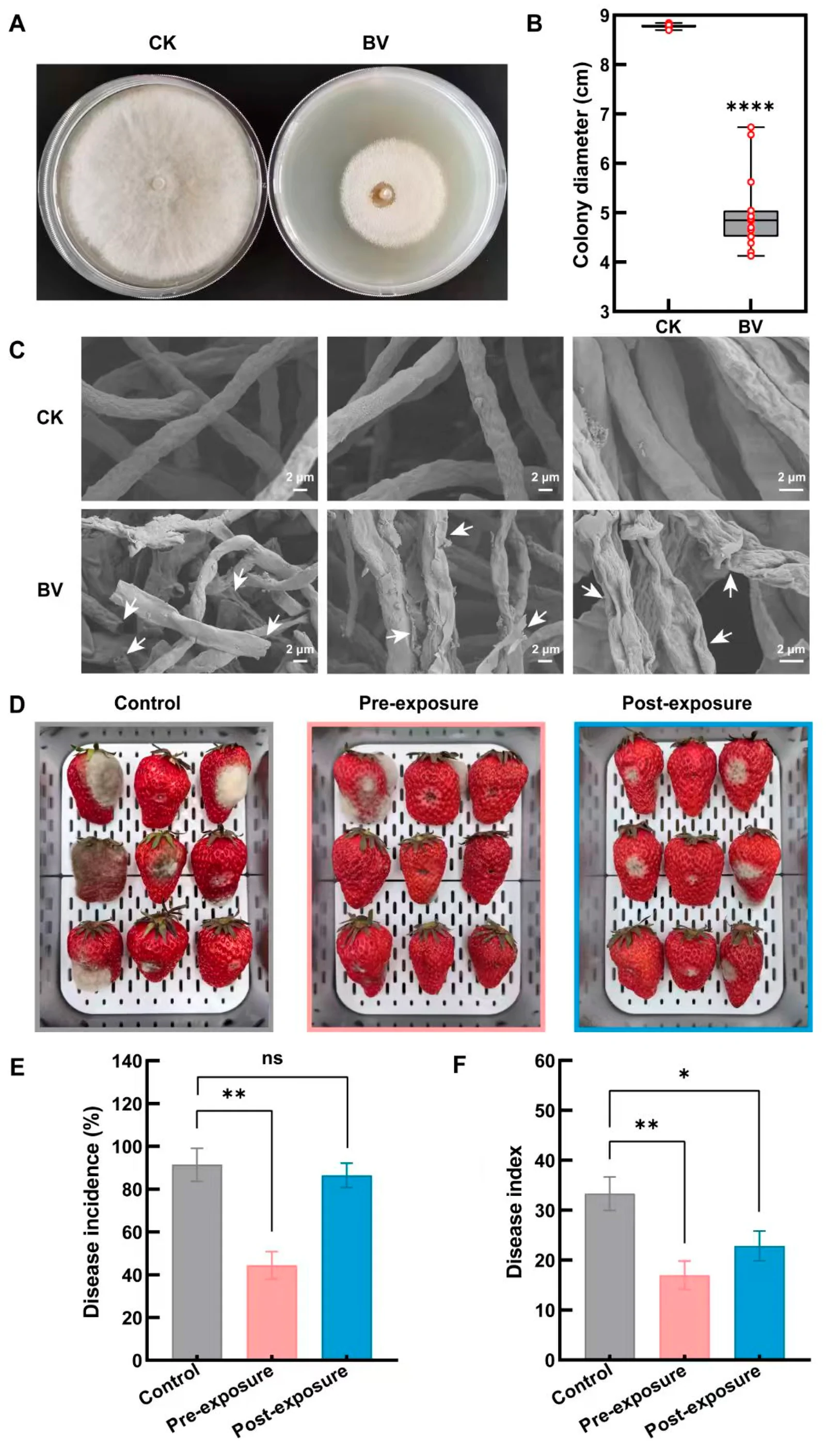
Inhibitory effect of *B. velezensis* CT32 VOCs (BV) on the growth of *B. cinerea* in vitro and in vivo. (**A**) BV suppressed the mycelial growth of *B. cinerea*. In the sealed plates test, fungus in the CK group showed normal growth on potato dextrose agar (PDA) medium. The mycelial growth of fungus in the BV group was suppressed by exposure to bacterial VOCs. (**B**) Colony diameter of *B. cinerea* without BV exposure or exposed to BV. (**C**) Scanning electron micrographs of *B. cinerea* hypha without BV exposure or exposed to BV, highlighting points of BV−induced hyphal damage (white arrow). (**D**) Gray mold symptoms on fruits of strawberry inoculated with unexposed mycelia (control); inoculated with mycelia pre−exposed to BV (pre−exposure); inoculated with mycelia and then exposed to BV (post−exposure). Fruits were shown at 5 days post inoculation (dpi). (**E**) Disease incidence and (**F**) disease index of strawberries inoculated with mycelia unexposed, pre−exposed or post−exposed to BV at 5 dpi. Error bars indicate standard deviation. Significance levels are denoted as * *p* < 0.05; ** *p* < 0.01; **** *p* < 0.0001; “ns” indicates no significant difference.

**Figure 2:**
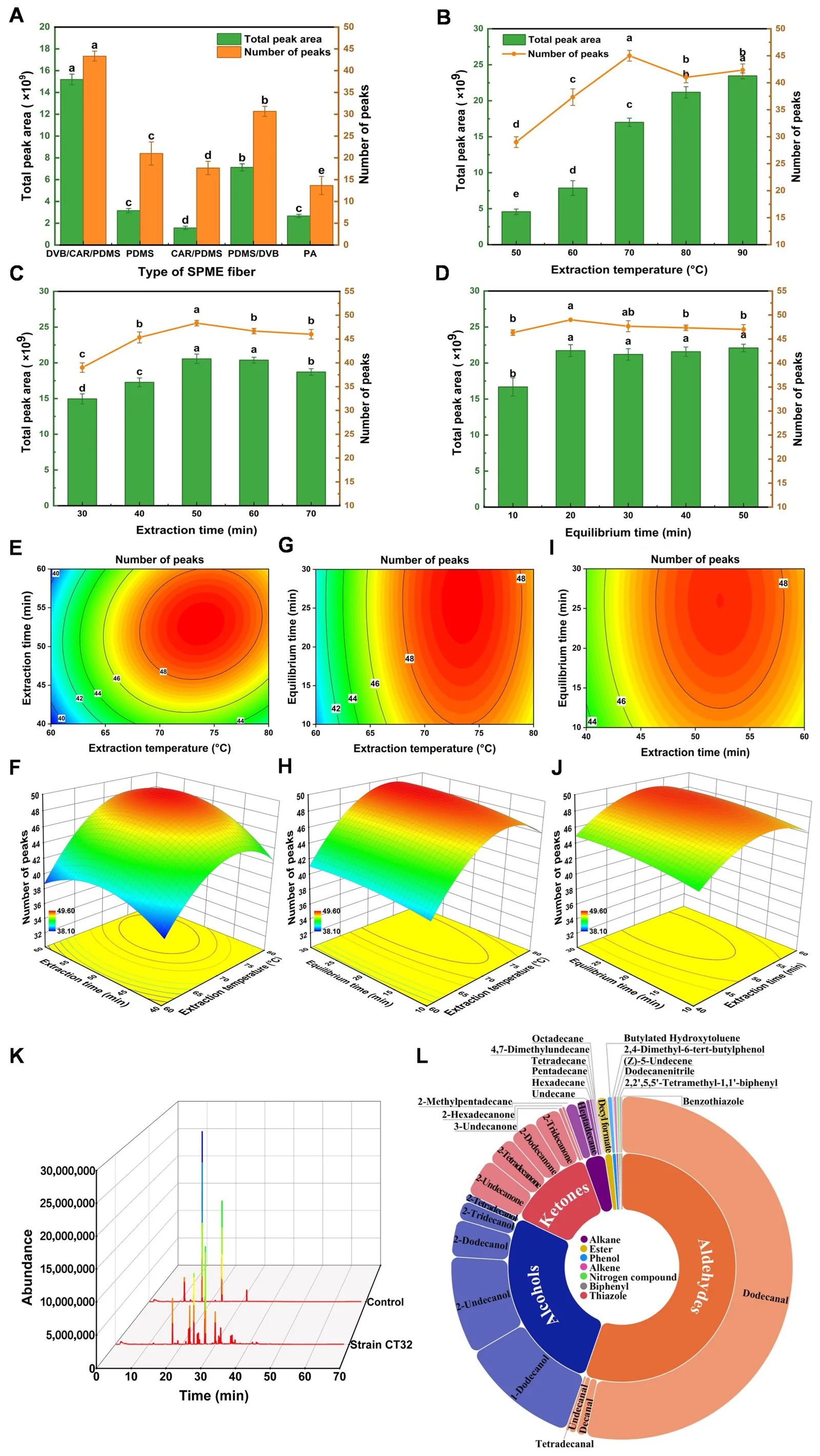
Identification of VOCs produced by strain CT32 using HS−SPME−GC−MS. Effect of (**A**) SPME fiber type, (**B**) extraction temperature, (**C**) extraction time, and (**D**) equilibrium time on the extraction efficiency of VOCs produced by strain CT32 expressed in terms of total peak area and number of peaks. 2D contour plots and their corresponding 3D response surface diagrams depicting the interactions between the variables affecting the amount of extracted compounds expressed as number of peaks: (**E**,**F**) extraction temperature and extraction time (keeping equilibrium time constant); (**G**,**H**) extraction temperature and equilibrium time (keeping extraction time constant); (**I**,**J**) extraction time and equilibrium time (keeping extraction temperature constant). (**K**) Total ion chromatograms of VOCs from strain CT32 and uninoculated medium control. (**L**) Sunburst chart depicting the chemical composition and relative abundance of VOCs identified in the headspace of strain CT32. Values with different lowercase letters are significantly different according to Duncan’s multiple range test at the 0.05 level.

**Figure 3:**
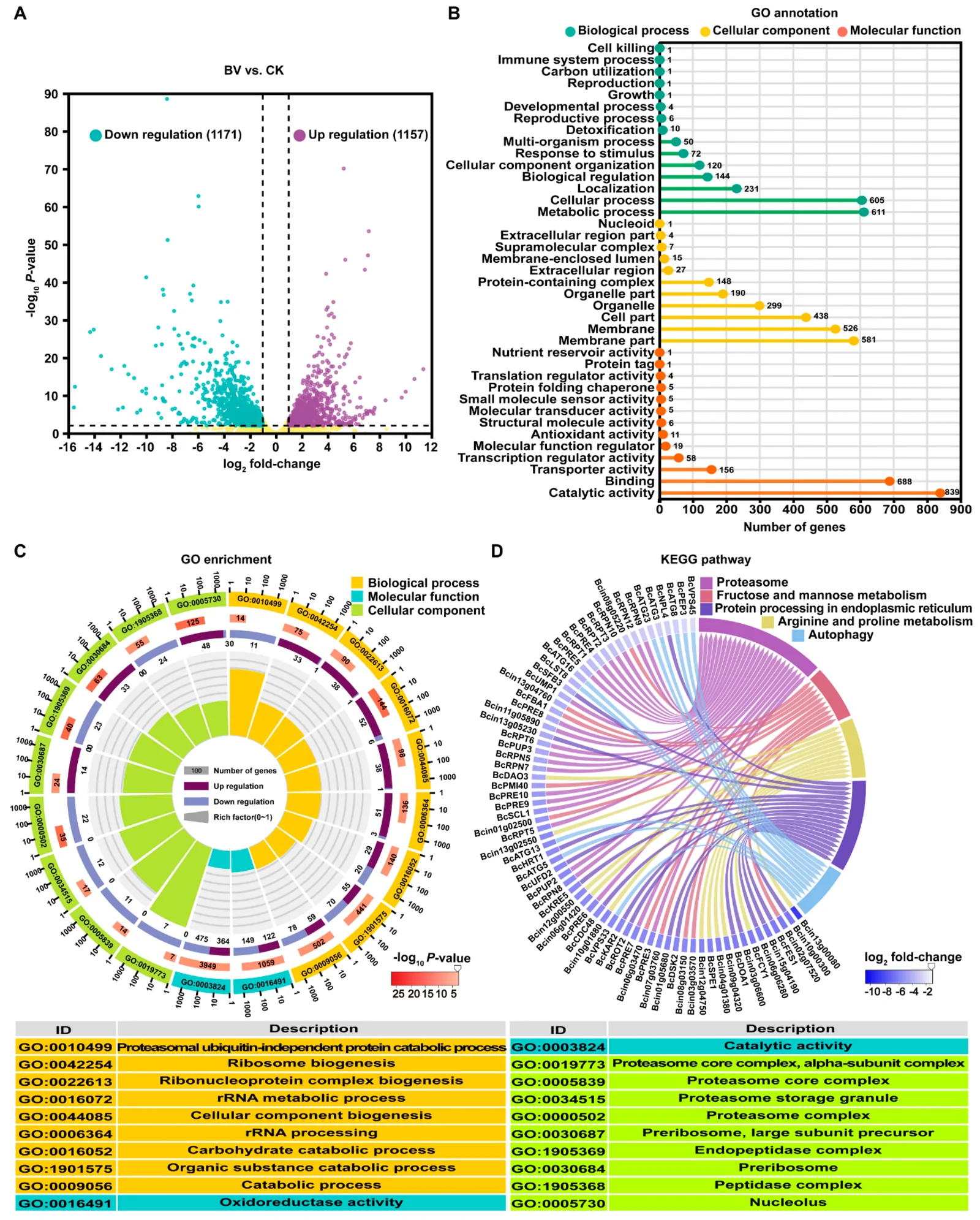
RNA−seq analysis reveals changes in gene expression profile of *B. cinerea* after exposure to BV. (**A**) Volcano plot of differentially expressed genes (DEGs) between BV group (mycelia exposed to strain CT32 VOCs) and CK group (mycelia not exposed to strain CT32 VOCs). (**B**) Matchstick chart of GO annotation for all DEGs. (**C**) Circle diagram of GO enrichment analysis. The outer circle represents the top 20 enriched GO terms. The second circle represents the number of genes annotated to a particular GO term and the adjusted *p*−value for the term. The third circle represents the number of upregulated (dark purple) and downregulated (light purple) genes in each corresponding GO term. The inner circle represents the rich factor. The higher the rich factor, the greater the degree of enrichment. (**D**) Chord diagram of KEGG pathway enrichment analysis of downregulated DEGs.

**Figure 4:**
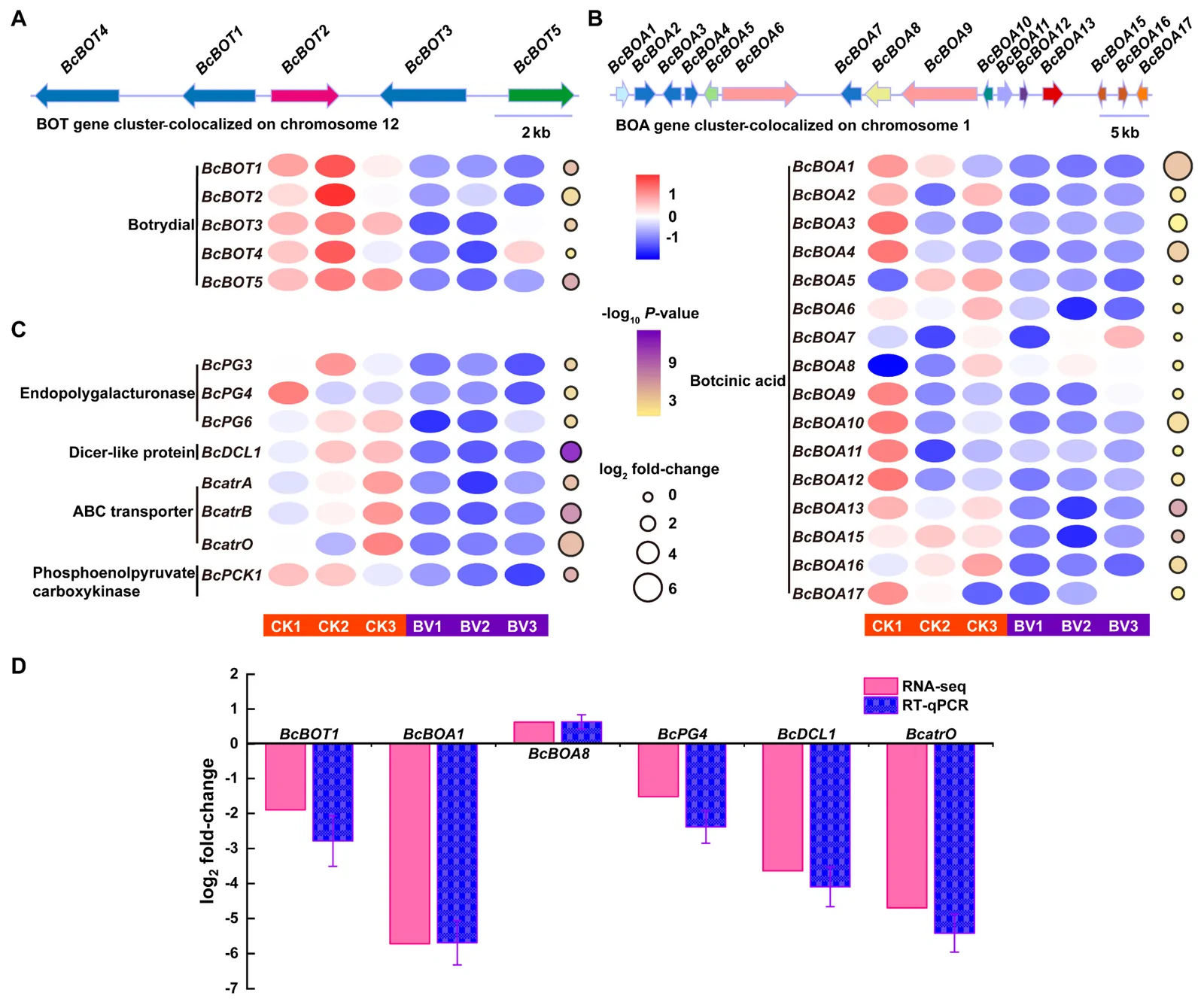
Differential gene expression following BV exposure reflects reduced pathogenicity of *B. cinerea*. Heatmap showing differential gene expression between CK group (mycelia not exposed to strain CT32 VOCs; CK1—CK3) and BV group (mycelia exposed to strain CT32 VOCs; BV1—BV3) of *B. cinerea*: (**A**) botrydial biosynthetic gene cluster; (**B**) botcinic acid biosynthetic gene cluster; (**C**) virulence−related genes. Red and blue shadings represent higher and lower relative expression levels, respectively. Circle color represents −log_10_ *p*−value and circle size represents log_2_ fold−change, which are used to measure relative expression between the two groups. (**D**) Comparison of log_2_ fold−change values for six pathogenicity−related genes as measured by RNA−seq and RT−qPCR. Error bars indicate standard deviation of the mean (*n* = 3).

**Figure 5:**
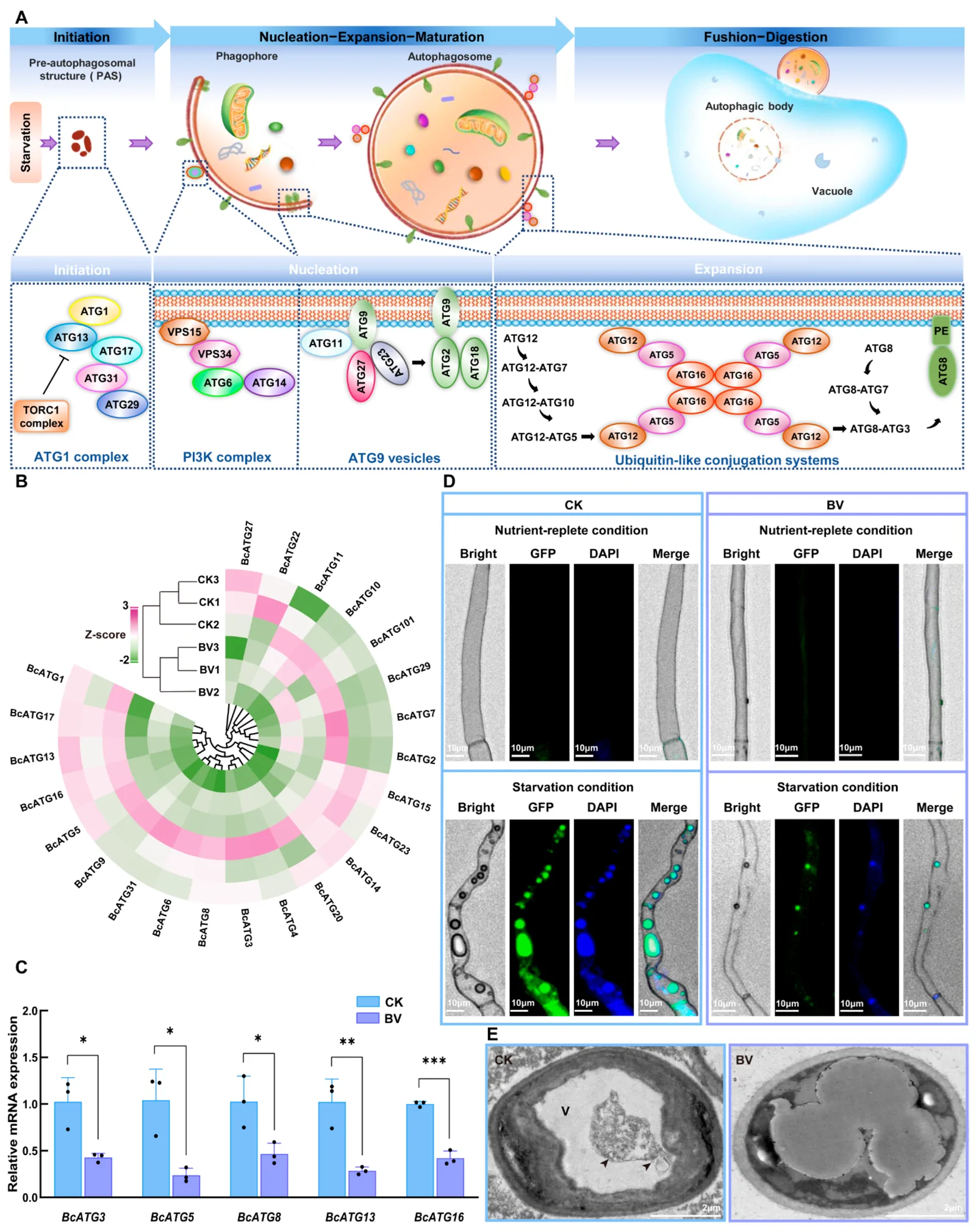
BV exposure−induced transcriptional repression of autophagy−related (ATG) genes results in impaired autophagy in *B. cinerea*. (**A**) Schematic illustration of autophagy process in *B. cinerea*. (**B**) Heatmap showing differential expression of ATG genes between CK group (mycelia not exposed to strain CT32 VOCs; CK1—CK3) and BV group (mycelia exposed to strain CT32 VOCs; BV1—BV3) of *B. cinerea*. Red and green shadings represent higher and lower relative expression levels, respectively. (**C**) Relative mRNA expression of five core genes involved in *B. cinerea* autophagy as determined by RT−qPCR. (**D**) Representative images of MDC staining in non−exposed or BV−exposed mycelia under nutrient−replete or starvation conditions. Nitrogen starvation induces accumulation of MDC−labeled vesicles in non−exposed control mycelia, while no such accumulation was observed in the BV−exposed mycelia. (**E**) Representative TEM images of autophagic structure in starved mycelia of *B. cinerea* not exposed or exposed to BV. Mycelia show signs of autophagy defect following exposure to BV. Black arrowhead: autophagic body; V: vacuole. Scale bar = 2 μm. Data are the mean ± standard deviation (*n* = 3). Significance levels are denoted as * *p* < 0.05; ** *p* < 0.01; *** *p* < 0.001.

**Table 1:** Analysis of variance (ANOVA) of the Box–Behnken design experiment for the extraction of VOCs by HS−SPME from strain CT32.

Source	Sum of Squares	Degree of Freedom	Mean Square	*F*-Value	*p*-Value
Model	226.21	9	25.13	113.51	<0.0001
*x*_1_-Extraction temperature	78.13	1	78.13	352.82	<0.0001
*x*_2_-Extraction time	12.50	1	12.50	56.45	0.0001
*x*_3_-Equilibrium time	3.13	1	3.13	14.11	0.0071
*x*_1_ *x*_2_	4.00	1	4.00	18.06	0.0038
*x*_1_ *x*_3_	0.25	1	0.25	1.13	0.3233
*x*_2_ *x*_3_	0.00	1	0.00	0.00	1.0000
*x*_1_^2^	86.21	1	86.21	389.35	<0.0001
*x*_2_^2^	32.42	1	32.42	146.43	<0.0001
*x*_3_^2^	1.16	1	1.16	5.24	0.0559
Residual	1.55	7	0.22		
Lack of fit	0.75	3	0.25	1.25	0.4028
Pure error	0.80	4	0.20		
Cor Total	227.76	16			
R^2^	0.9932				
Adjusted R^2^	0.9844				

## Data Availability

The RNA-seq data have been deposited in the NCBI’s Sequence Read Archive (SRA) under the accession number: PRJNA1171356.

## Supplementary Materials

The following supporting information can be downloaded at https://www.mdpi.com/article/10.3390/jof12100731/s1. Figure S1: Schematic diagram of experimental procedure for VOC profiling of strain CT32; Table S1: Gene-specific primers used in the RT-qPCR analysis; Table S2: Design matrix and experimental results of the Box–Behnken experiment; Table S3: Chemical composition of VOCs released by *Bacillus velezensis* CT32; Table S4: Summary statistics of RNA-seq data and mapping results.
