# Supplementary material for: Volatile Organic Compounds Produced by Bacillus velezensis CT32 Attenuate Botrytis cinerea Pathogenicity by Impairing Autophagy
Source: J Fungi (Basel). 2026 Oct 1;12(10):731. doi: 10.3390/jof12100731 (PMC13706159; doi:10.3390/jof12100731)
Supplement: Supplementary file 1 [file jof-12-00731-s001.zip › Supplementary material.pdf]

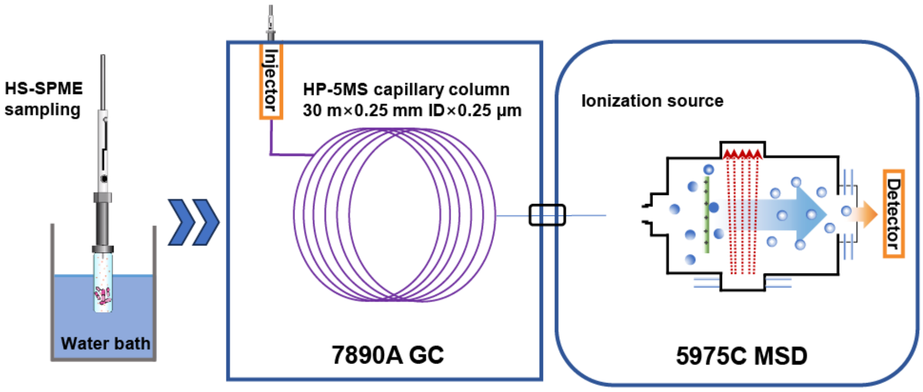

Figure S1. Schematic diagram of experimental procedure for VOC profiling of strain CT32.

Table S1. Gene-specific primers used in the RT-qPCR analysis.

Gene name	Primer sequences (5'→3')
BcBOT1	F: CCTACCTCACCTTCTCTAACTTCTTC<br>R: CCTTGACTCTGACATGCTTCTTAATC
BcBOA1	F: ACATTCCCAGCAGAACCTCG<br>R: GACTGTCGGTCCCCAACAAT
BcBOA8	F: TGATGCGGTCTCGGAAAACA<br>R: ATCGGCGCATCAGTTTCAGA
BcPG4	F: GCTTCCGACACTGTTCTTATTGAG<br>R: AGATAATGTTGCTTCCAGAGTTGATAG
BcDCL1	F: TTCCCTTGCTAAGACTGCGG<br>R: GCTTCAGGACCTGGCATCTT
BcatrO	F: CGTTCTATACGAGGGCCACC<br>R: GCAAACTCACTCGCGGTTTT
BcATG3	F: AGGATGATGACGATGACGATGAAG<br>R: AGGAGTTCTGTAATAGGGTGTATATGC
BcATG5	F: CTATTCTCCCAAGTTTATTTCCAAGTAGG<br>R: CCATCCGCATAACTCGCTTCC
BcATG8	F: TAAGGACGAGCACCCATTCG<br>R: TATTTTTGACGGATGCGTTCAG
BcATG13	F: GAGTCCAATATCCGCCTTGAGAG<br>R: GAGTAATGGTCGTGATTGTGGTAAC
BcATG16	F: CCAAAAGTGCTTCGGTGTCCA<br>R: TTTAGCTCTCAACTCCTCGTCT
BcactA	F: ATCTTGGCTGAGCGTGGTTAC<br>R: GACTGGCGGTTTGGATTTCTTG

Table S2. Design matrix and experimental results of the Box-Behnken experiment.

Run	Extraction temperature (°C)		Extraction time (min)		Equilibrium time (min)		Number of peaks
	x_1 (coded)	X_1 (uncoded)	x_2 (coded)	X_2 (uncoded)	x_3 (coded)	X_3 (uncoded)	Y (observed)
1	0	70	0	50	0	20	49
2	−1	60	0	50	1	30	41
3	0	70	0	50	0	20	49
4	−1	60	1	60	0	20	39
5	0	70	0	50	0	20	48
6	0	70	−1	40	1	30	45
7	−1	60	0	50	−1	10	40
8	1	80	0	50	1	30	48
9	1	80	−1	40	0	20	42
10	0	70	1	60	−1	10	46
11	1	80	1	60	0	20	47
12	0	70	−1	40	−1	10	44
13	1	80	0	50	−1	10	46
14	−1	60	−1	40	0	20	38
15	0	70	0	50	0	20	49
16	0	70	0	50	0	20	49
17	0	70	1	60	1	30	47

Table S3. Chemical composition of VOCs released by *Bacillus velezensis* CT32.

Retention time (min)	Compound	CAS number	Molecular formula	Group	Similarity (%)	Determined retention index	Retention index from reference	Relative abundance (%)
12.40	2-Ethyl-1-hexanol	104-76-7	$C_8H_{18}O$	Alcohol	90	1031.93	1031 [1]	0.00 ± 0.00 [2]
14.70	(Z)-5-Undecene	764-96-5	$C_{11}H_{22}$	Alkene	93	1094.06	–	0.28 ± 0.05
14.95	Undecane	1120-21-4	$C_{11}H_{24}$	Alkane	93	1100.89	1100	0.34 ± 0.13
17.48	– [3]	–	–	–	–	–	–	0.18 ± 0.03
17.81	–	–	–	–	–	–	–	0.42 ± 0.04
18.12	–	–	–	–	–	–	–	0.24 ± 0.03
18.21	–	–	–	–	–	–	–	0.28 ± 0.04
18.57	–	–	–	–	–	–	–	0.38 ± 0.04
18.74	Decanal	112-31-2	$C_{10}H_{20}O$	Aldehyde	97	1206.04	1206	0.88 ± 0.13
19.50	Benzothiazole	95-16-9	C_7H_5NS	Thiazole	91	1227.13	1223	0.06 ± 0.01
21.12	Decyl formate	5451-52-5	$C_{11}H_{22}O_2$	Ester	86	1271.47	–	0.98 ± 0.09
21.31	4,7-Dimethylundecane	17301-32-5	$C_{13}H_{28}$	Alkane	86	1276.65	–	0.09 ± 0.07
21.74	3-Undecanone	2216-87-7	$C_{11}H_{22}O$	Ketone	90	1288.56	1283	0.34 ± 0.12
21.95	2-Undecanone	112-12-9	$C_{11}H_{22}O$	Ketone	94	1294.35	1294	3.37 ± 0.35
22.29	2-Undecanol	1653-30-1	$C_{11}H_{24}O$	Alcohol	90	1303.13	1303	8.46 ± 0.46
22.47	Undecanal	112-44-7	$C_{11}H_{22}O$	Aldehyde	95	1307.32	1307	0.60 ± 0.08
24.57	2-Dodecanone	6175-49-1	$C_{12}H_{24}O$	Ketone	90	1357.06	1348	2.43 ± 0.11
24.88	2-Dodecanol	10203-28-8	$C_{12}H_{26}O$	Alcohol	90	1364.50	1417	3.24 ± 0.09
25.19	–	–	–	–	–	–	–	1.37 ± 0.01
25.65	–	–	–	–	–	–	–	0.20 ± 0.04

25.87	2,4-Dimethyl-6-tert-butylphenol	1879-09-0	$C_{12}H_{18}O$	Phenol	90	1387.83	–	0.10 ± 0.01
26.38	Tetradecane	629-59-4	$C_{14}H_{30}$	Alkane	93	1399.98	1400	0.11 ± 0.02
26.98	Dodecanal	112-54-9	$C_{12}H_{24}O$	Aldehyde	87	1412.45	1411	48.56 ± 0.56
28.38	–	–	–	–	–	–	–	0.09 ± 0.02
29.12	–	–	–	–	–	–	–	0.51 ± 0.08
29.45	–	–	–	–	–	–	–	0.27 ± 0.04
29.93	1-Dodecanol	112-53-8	$C_{12}H_{26}O$	Alcohol	91	1474.06	1475	10.27 ± 0.20
30.17	–	–	–	–	–	–	–	0.82 ± 0.07
30.61	Dodecanenitrile	2437-25-4	$C_{12}H_{23}N$	Nitrogen compound	93	1488.39	1486	0.23 ± 0.05
30.98	2-Tridecanone	593-08-8	$C_{13}H_{26}O$	Ketone	95	1496.12	1496	2.34 ± 0.03
31.17	Pentadecane	629-62-9	$C_{15}H_{32}$	Alkane	93	1500.02	1500	0.11 ± 0.02
31.34	2-Tridecanol	1653-31-2	$C_{13}H_{28}O$	Alcohol	90	1503.08	1510	1.69 ± 0.20
31.83	Butylated Hydroxytoluene	128-37-0	$C_{15}H_{24}O$	Phenol	98	1511.78	1511	0.41 ± 0.10
34.56	2-Tetradecanone	2345-27-9	$C_{14}H_{28}O$	Ketone	94	1560.41	1597	2.56 ± 0.10
34.96	–	–	–	–	–	–	–	2.96 ± 0.14
35.31	2-Tetradecanol	4706-81-4	$C_{14}H_{30}O$	Alcohol	90	1573.82	1575	0.65 ± 0.05
35.85	2-Methylpentadecane	1560-93-6	$C_{16}H_{34}$	Alkane	83	1583.36	1569	1.05 ± 0.22
36.78	Hexadecane	544-76-3	$C_{16}H_{34}$	Alkane	96	1599.91	1600	0.19 ± 0.03
37.43	Tetradecanal	124-25-4	$C_{14}H_{28}O$	Aldehyde	91	1611.71	1611	0.28 ± 0.04
38.99	–	–	–	–	–	–	–	0.08 ± 0.00
39.45	–	–	–	–	–	–	–	0.19 ± 0.02
39.81	–	–	–	–	–	–	–	0.10 ± 0.00
40.61	–	–	–	–	–	–	–	0.13 ± 0.05

40.73	2,2',5,5'-Tetramethyl-1,1'-biphenyl	3075-84-1	$C_{16}H_{18}$	biphenyl	95	1671.99	1669	0.17 ± 0.06
42.27	Heptadecane	629-78-7	$C_{17}H_{36}$	Alkane	97	1699.93	1700	0.81 ± 0.27
42.66	–	–	–	–	–	–	–	0.49 ± 0.09
42.73	–	–	–	–	–	–	–	0.29 ± 0.08
45.89	–	–	–	–	–	–	–	0.16 ± 0.00
46.29	2-Hexadecanone	18787-63-8	$C_{16}H_{32}O$	Ketone	93	1772.03	1800	0.24 ± 0.04
47.85	Octadecane	593-45-3	$C_{18}H_{38}$	Alkane	96	1799.80	1800	0.05 ± 0.01

[1] The reference value was obtained from the NIST Chemistry WebBook.

[2] Data are presented as mean ± standard deviation of three independent HS-SPME-GC-MS analyses.

[3] "–" denotes an unidentified compound.

Table S4. Summary statistics of RNA-seq data and mapping results.

Sample	Repetition	Raw reads	Clean reads	Clean bases	GC content (%)	Q30 (%)	Mapped reads	Mapped reads ratio (%)
CK control	1	45344434	45088592	6692773043	45.90	94.81	29466581	65.35
	2	41603124	41329754	6197219111	46.02	94.39	28505819	68.97
	3	40584366	40295648	6005626833	46.07	95.02	30071382	74.63
BV treatment	1	41009460	40763564	6116545905	46.50	94.94	39022412	95.73
	2	49112884	48772258	7308098791	46.84	94.72	46580364	95.51
	3	44266180	43889802	6557200292	46.65	94.83	42150457	96.04
